# Aging and activity patterns: actigraphy evidence from NHANES studies

**DOI:** 10.3389/fsysb.2025.1632110

**Published:** 2025-10-14

**Authors:** Wen Luo, Matthew T. Scharf, Ioannis P. Androulakis

**Affiliations:** ^1^ Biomedical Engineering Department, Rutgers University, New Brunswick, NJ, United States; ^2^ Division of Sleep Medicine, Department of Neurology, Hackensack Meridian Health, Neptune, NJ, United States

**Keywords:** actigraphy, chronotype, aging, sleep-wake cycle, physical activity, circadian rhythms, NHANES, machine learning

## Abstract

**Study objectives:**

This study examines age-related variations in activity patterns using actigraphy data from the National Health and Nutrition Examination Survey (NHANES). By analyzing sleep onset, wake times, and daily activity levels across different age groups, we aim to uncover key changes in chronotype and physical engagement with aging. From a systems-biology perspective, minute-level rest–activity traces are emergent outputs of coupled circadian–homeostatic–behavioral networks. Treating actigraphy as a high-throughput phenotyping readout, we use NHANES to extract system-level markers (phase, amplitude, and transition dynamics) that reflect network organization across the lifespan.

**Methods:**

Actigraphy data from NHANES (2011–2013) were analyzed using machine learning techniques to identify distinct activity clusters among four age groups (19–30, 31–50, 51–70, 71–80). We implemented an unsupervised machine learning pipeline that clustered average-day actigraphy profiles, enabling the identification of distinct, age-dependent rest–activity phenotypes from the NHANES dataset. Sleep-wake cycles, activity intensities, and circadian periodicities were assessed through clustering and statistical modeling. Key metrics, including winding down activity and time to alertness, were derived to evaluate age-related variations.

**Results:**

Younger individuals exhibited delayed chronotypes with later sleep and wake times, whereas older adults showed advanced and more structured schedules. Winding down periods lengthened with age, and overall activity levels declined progressively. Time to alertness showed a strong correlation with wake time in younger groups but diminished with age, indicating a weakening circadian influence.

**Conclusion:**

Aging is associated with shifts in sleep-wake cycles and activity patterns, reflecting biological and behavioral adaptations. These findings highlight the importance of personalized interventions to support optimal activity and sleep alignment across the lifespan. Insights from actigraphy data can inform public health strategies and clinical approaches to aging-related changes in physical activity and circadian regulation. These age-stratified, interpretable “dynamical phenotypes” provide observables to calibrate and validate systems-level models of sleep–wake regulation and behavior–physiology coupling, supporting hypothesis generation and intervention design in systems biology.

## Introduction

Understanding a person’s activity patterns is crucial for health, wellbeing, and productivity. Activity patterns reflect the timing, intensity, and regularity of physical movements and behaviors throughout the day, providing insights into an individual’s lifestyle, habits, and overall functioning (Sani et al., 2015; Mitchell et al., 2017). Researchers and clinicians can identify health risks by analyzing activity patterns, as disruptions like sedentary behavior or irregular routines contribute to chronic diseases. In circadian biology, misalignment between biological rhythms and daily schedules (e.g., shift work, social jetlag) can impair sleep, cognition, and mental health. In clinical settings, monitoring activity patterns can assist in diagnosing conditions such as sleep disorders, depression, or circadian rhythm disorders (Liguori et al., 2023; Hashimoto et al., 2015). Understanding activity patterns enables personalized interventions, optimizing exercise, behavior, and rest for better health and quality of life. Societally, these insights inform public policies that promote physical activity, enhance productivity, and reduce lifestyle-related illnesses, fostering healthier, more sustainable lifestyles. Appreciation of a person’s activity patterns is particularly important in the context of aging, as these patterns undergo changes with advancing age and provide critical insights into health and functional status (Stenholm et al., 2021). As people age, physical activity declines, and daily patterns become less structured due to biological changes, chronic conditions, and social factors. Monitoring activity in older adults can signal declining physical function, frailty, or cognitive decline, helping predict risks like falls, cardiovascular disease, and loss of independence.

Actigraphy is a non-invasive method for recording activity patterns by measuring wrist movement with a small motion sensor called an accelerometer, offering a window into how external factors like work, social demands, or environmental light influence activity timing (Gao et al., 2023). Actigraphy is widely used in sleep research due to its convenience, affordability, and ability to collect data over long durations in real-world settings (Fekedulegn et al., 2020). Actigraphy objectively tracks sleep and activity, reducing recall bias and accurately identifying chronotypes (chronotype: an individual’s characteristic timing of sleep–wake and daily activity, reflecting the phase of their endogenous circadian system relative to the 24-h day.) By analyzing sleep timing, duration, and variability, researchers classify individuals as morning or evening chronotypes. Beyond sleep, actigraphy reveals daily activity rhythms, reflecting energy levels and preferences. Of particular importance are studies that assess variations in a patient’s activity levels throughout the day in the presence of disease (Hashimoto et al., 2015; Kos et al., 2007; George et al., 2021; Nikbakhtian et al., 2021; Schneider et al., 2022). Actigraphy generates vast data, often leading to storage, processing, and analysis challenges. Several computational approaches have been proposed for the statistical analysis of physical activity based on actigraphy (Zhang et al., 2019; Krafty et al., 2019; Roberts et al., 2023), which is a subset of a more significant problem focusing on the analysis of time series data (Bagnall et al., 2017).

Understanding age-related rest–activity patterns at population scale is limited by (i) reliance on pre-specified sleep windows or questionnaire chronotype, (ii) single-metric summaries that obscure multimodal behavior, (iii) small or device-specific cohorts, and (iv) minimal, age-specific normative references derived from minute-level data. We address these gaps with a scalable, unsupervised pipeline that (1) filters for robust 24-h periodicity, (2) compresses minute-level NHANES MIMS data into an interpretable “average-day” profile, (3) performs age-stratified clustering to reveal behavioral phenotypes, and (4) derives simple, clinically legible markers—sleep onset and wake proxies from a piece-wise constant model, winding-down time/activity, and time-to-alertness. This framework yields age-specific normative distributions from a nationally representative sample and provides transparent, reproducible endpoints for research and practice.

In the present work, we analyzed actigraphy data from the U.S. National Health and Nutrition Examination Survey (NHANES) database, encompassing over 10,000 individuals, and applied unsupervised clustering to uncover distinct activity patterns as a function of age. In NHANES 2011–2014, participants wore wrist-worn ActiGraph GT3X + accelerometers continuously for 7 days, enabling analysis of minute-level rest–activity rhythms. Earlier NHANES cycles (2003–2006) used hip-worn ActiGraph AM-7164 accelerometers primarily for physical activity assessment. A simple yet informative methodology facilitated by machine learning techniques identified several distinct age-dependent patterns characterized by activity onset, resolution, and intensity variations. Our age-dependent analysis revealed biases towards specific age groups within these clusters, underscoring the relationship between age and chronotype. Notably, younger clusters exhibited delayed chronotypes with significant differences in sleep onset time (SOT) and wake time (WT) compared to older clusters, suggesting a phase advance in sleep patterns with age. Additionally, the clusters displayed distinct patterns in winding up and winding down periods, providing valuable insights into the dynamics of activity transitions. This study demonstrates that efficient processing of large-scale actigraphy data enables robust chronotype characterization which can inform personalized healthcare and public health initiatives.

In systems biology, observable behaviors such as sleep–wake timing and diurnal activity arise from interacting control loops, circadian pacemakers, homeostatic sleep drive, endocrine and autonomic modulators, and social/lighting inputs. We therefore treat minute-level MIMS actigraphy as a behavioral “omics” signal and compress it into an average-day macro-observable on which we perform unsupervised phenotyping. The derived markers,sleep onset/wake proxies, winding-down time/activity, and time-to-alertness, map onto classical systems concepts (phase, gain/amplitude, and transition kinetics), offering population-scale constraints for mechanistic models (e.g., coupled ODEs of sleep–wake regulation and entrainment). In short, our pipeline links wearable data to system-level parameters, enabling integrative studies of aging as progressive reparameterization of the underlying regulatory network.

## Methods

### Data

We extracted physical activity monitoring data from the NHANES database. Specifically, we considered recordings from the2011^1^ and2013^2^ surveys. The actigraphy data are provided per minute (variable name: PAXMTSM). The nationwide, cross-sectional surveys contained recordings for 6,710 (2011) and 7,401 (2013) individuals, respectively. Thus, our study considered 14,111 recordings of actigraphy data over 7 days. Not all individuals had complete records. However, our approach considers an average day, so missing data were averaged. The collection process details are standardized and have already been described in earlier publications (Shim et al., 2023; Su et al., 2022).

Our study examines age-dependent changes in adult activity patterns by dividing participants into four age groups: 19–30, 31–50, 51–70, and 71–80. Individuals under 19 were excluded because sleep–wake timing and activity patterns change markedly through childhood and adolescence (e.g., pubertal phase delay, different sleep needs, and strong weekday–weekend swings driven by school schedules), while adults, with more structured routines shaped by work, family, and social obligations, provide more reliable comparisons. These divisions align with widely accepted classifications (Geifman et al., 2013), such as those in the Medical Subject Headings (MeSH, http://www.ncbi.nlm.nih.gov/mesh), ensuring relevance to distinct life stages and behavioral patterns.

• Young Adulthood (19–30): Characterized by high physical fitness, diverse activities, and flexible routines influenced by education, work, and social life. Activity patterns vary widely, with irregular sleep-wake cycles common.
• Mid-Adulthood (31–50): Defined by structured routines shaped by career and family, reduced leisure activity, and emerging age-related declines. Actigraphy reveals the impact of work, caregiving, and physiological changes on movement.
• Older Adulthood (51–70): Marked by retirement, shifting activity patterns, increased chronic conditions, and sleep fragmentation. While mobility declines, some remain active through recreation and exercise, making actigraphy crucial for assessing aging’s impact.
• Senior Years (71–80): Characterized by increased sedentary behavior, reduced physical activity, and reliance on assistive devices due to frailty and chronic conditions. With stable routines, actigraphy guides interventions to promote mobility and quality of life.

### Analysis

Activity data are recorded at 1 min resolution, and the wearable devices are used for a maximum of 7 days. The NHANES Survey objectively measures physical activity using a Physical Activity Monitor (PAM). This device recorded acceleration across three axes (x, y, and z) at a frequency of 80 Hz, along with ambient light levels at 1 Hz. In the NHANES dataset, PAXMTSM represents each minute’s MIMS (Motion Intensity Measurement Summary) triaxial value. It is a summary measure derived from accelerometer data collected by physical activity monitors (PAMs) worn by participants. The PAXMTSM variable is calculated by summing the minute summary acceleration measurements obtained on the x-, y-, and z-axes (PAXMXM, PAXMYM, and PAXMZM). It represents the total movement intensity recorded during that minute. NHANES provides these MIMS variables directly, so no additional processing of raw accelerometer signals was required for our analyses. We include the NHANES variable name to enable readers to locate the exact field in the public database. A plethora of methods exist for analyzing longitudinal data, including actograms (Shim et al., 2023). Our analysis aimed at describing succinctly broad characteristics of groups of individuals.

**FIGURE 1:**
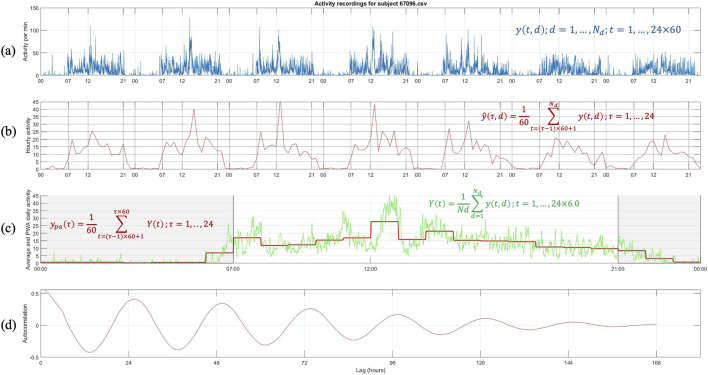
Steps for the representation and analysis of an actogram. **(a)** Raw activity data recorded per minute for a subject across multiple days, denoted as $y\left(t,d\right),$ where dd is the day and tt is the minute of observation. **(b)** Hourly activity averages, $y^{\prime }\left(\tau ,d\right)$ computed by aggregating minute-level activity within each hour (τ = 1, … ,24). **(c)** Daily activity profiles, derived by averaging across all days, showing both raw ( $Y\left(t\right)$ , green line) and hourly averaged data ( $y_{pa}\left(\tau \right),$ red line), highlighting daily activity patterns. **(d)** Autocorrelation of the activity data over multiple lags (hours), revealing periodicities and rhythms in the activity patterns. These steps illustrate the processing pipeline for creating a representative actogram and analyzing further circadian activity trends.

Data selection and representation: The raw data (Figure 1a) is reshaped into a three-dimensional matrix representing minutes, hours, and days. Hourly averages are then computed by taking the mean of the minute-level data for each hour, producing a compact representation of the activity pattern as a 7-day by 24-h matrix (Figure 1b). This step reduces the dimensionality of the data and highlights broader patterns in daily activity. To identify intrinsic periodicities, the hourly-averaged activity data are subjected to autocorrelation analysis (Figure 1d). The autocorrelation function (ACF) is calculated to measure the similarity of the activity signal at different time lags, up to 168 h (the total hours in 7 days). Peaks in the smoothed ACF represent recurring patterns, and the time intervals between successive peaks (lags) are extracted to estimate the dominant periodicity. These intervals indicate how often activity patterns repeat, with periodic peaks around 24 h suggesting circadian alignment. The estimated periodicities are statistically evaluated using a chi-square test to determine their alignment with the expected 24-h cycle. The null hypothesis assumes the periods are centered around 24 h. A $p_{val}$ is calculated to assess the likelihood of observing the detected periodicity under this assumption. If the $p_{val}$ exceeds a threshold (e.g., 0.01), the data are considered consistent with a 24-h rhythm; otherwise, it is classified as inconsistent. The results guide the classification of subjects: data demonstrating consistent 24-h periodicity is accepted for further analysis, while inconsistent data are excluded. This rigorous approach ensures the identification of intrinsic rhythms that align with circadian patterns, providing robust insights into the periodic behavior of activity data.

The recordings are reported at the rate of one per minute, therefore, there are 24×60=1440 daily samples, one for each minute of the 24-h day. Subsequently, (Figure 1c), the recording at each minute of the day is averaged over the number of days for which recordings exist, $Y\left(t\right)=\frac{1}{Nd}\sum_{d=1}^{N_{d}}y\left(t,d\right);t=1,\ldots ,24\times 6\;0$ . We do so because we treat each day as a replicate. In doing so, we may lose day-to-day variations. However, because of the short timespan for recordings, it is hard to differentiate between errors and persistent patterns (such as shift work). The method would still be valid without losing generality if one considered daily variations (this issue will be further discussed later.) Finally, the per-minute average day’s activity profile is transformed using a piece-wise constant approximation (PCA) to generate an hourly average activity profile $y_{pa}\left(\tau \right)=\frac{1}{60}\sum_{t=\left(\tau -1\right)\times 60+1\;}^{\tau \times 60}Y\left(t\right);\tau =1,..,24$ , Figure 1c. The piece-wise constant representation of the average day of each subject is used for further analyses.

**FIGURE 2:**
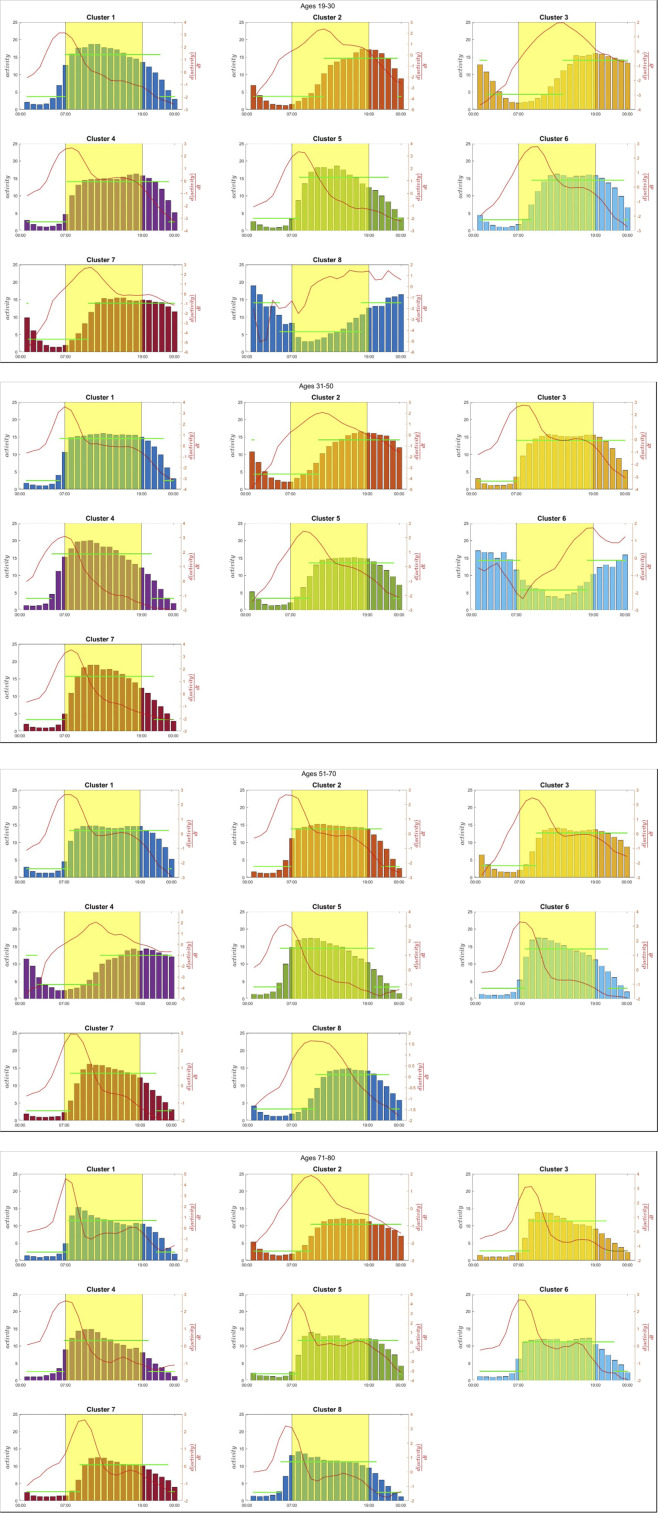
Cluster center activity profiles for all age groups represented on an hourly basis. Each subpanel corresponds to a specific cluster and shows the hourly activity levels (bar plots), the derivative of activity (red line), and the linear approximation of cluster center activity trends (green line). The shaded yellow region indicates typical daytime period. These profiles highlight distinct activity patterns across clusters and age groups, emphasizing variations in activity intensity, temporal distribution, and transitions between active and resting states.

Clustering and piece-wise representation of cluster centers: Once the subjects’ actograms have been approximated as described earlier, the profiles are clustered using Kmeans with the center clusters depicted in Figure 2.

Each center’s cluster is approximated by a piece-wise contsant function (Equation 1; Figure 2) such that:

$$PLA\left(t\right)=\left\{\begin{array}{c} \alpha ,SOT\leq t\leq WT\;\\ \beta ,t\leq SOT\vee t\geq WT \end{array}\right.$$ (1)

We also compute the derivative of the activity level over time for each cluster center (Figure 2). The derivative of the activity of the cluster center is an indication of the direction of the subject’s activity levels: a positive derivative indicates the period of the day during which the person is engaged in increased activity levels, whereas a negative implies the opposite. The derivative, along with the activity values over time of the cluster center, will be used to calculate some essential quantities, which will be discussed in greater detail in the next section.

## Results

Combining the 2011–2013 data created a cohort of 10,016 individuals for which 7 days of actigraphy and age data were available. The data represent a 50/50 split between males and females, ranging in age from 12 to 80, with a median age of 41. Considering individuals older than nineteen, 4,775 subjects were found to have actograms with a leading periodicity of 24 with a high level of confidence. The remaining actograms were of relatively poor quality and unsuitable for further analysis. The final set had 938 subjects aged 19–30; 1,633 subjects aged 31–50; 1,576 subjects aged 51–70; and 628 subjects aged 71–80. Each actogram was analyzed as previously described, and the “per minute” activity data were combined to generate an “average” day for each subject. To estimate the optimal number of clusters, we employed a data-driven approach based on analyzing the within-cluster sum of squares (WCSS) across a range of cluster counts. Specifically, we computed the first and second derivatives of the WCSS curve to identify the point where the rate of improvement in clustering performance begins to level off. The optimal number of clusters is selected as the value of *k* just before the second derivative reaches its minimum, which corresponds to the point where the curve becomes flattest, i.e., where adding more clusters yields diminishing returns. While this method does not guarantee identification of the visually apparent “elbow” in all cases, it offers an objective and reproducible way to detect stabilization in cluster compactness. This is particularly valuable when the elbow is not sharply defined. Although some sensitivity to noise or gradual curves may occur, the approach is grounded in the underlying curvature of the WCSS trajectory and provides a mathematically coherent criterion for model selection. As such, it represents a reasonable and justifiable method for estimating the optimal number of clusters, especially in exploratory analyses where fully automated guidance is desirable. Each group was clustered separately, and the results are shown in Figure 2 for all age groups. The figure depicts each cluster center’s per-hour activity levels in the form of bar graphs. The corresponding PCA is also shown for each cluster center. The parameters for each fit in (1) are presented in Table 1. In this approximation, we loosely define sleep onset time (SOT) and wake time (WT) as the beginning and end of the lower activity periods based on the piece-wise constant approximation. The left axis (bars) depicts the hourly activity of each cluster center: $activity\left(t\right),t=1,\ldots ,24$ , whereas the right axis depicts (red line) the derivative of the cluster center activity: $\frac{d\left[activity\left(t\right)\right]}{dt},t=1,..,24$ . The derivatives are calculated using 4^th^-order finite difference approximation for the interior points and lower order for the endpoints. The derivatives are calculated to determine periods of the day during which the subjects exhibit increases and decreases in activity levels.

**TABLE 1:** Comparison of SOW, WT, α, and β metrics across different age groups (19–30, 31–50, 51–70, and 71–80) with corresponding counts.

Age group 19-30				
SOT	WT	α	β	count
21.71	6.92	3.72	15.71	110
23.42	11.97	3.75	14.67	98
2.03	13.93	14.09	4.35	78
23.05	7.23	2.54	14.07	196
22	8.18	3.55	15.31	109
23.37	9.07	3.12	14.48	191
1.16	10.55	13.95	3.66	142
5.06	17.82	14.12	5.83	14

Age group 31-50				
SOT	WT	α	β	count
22.41	6.24	2.5	14.55	362
1.28	11.4	14.18	4.36	154
23.99	7.08	2.33	14.03	397
20.5	5	3.35	16.18	167
23.14	9.93	3.4	13.58	245
7.56	18.08	14.32	5.77	13
20.94	7.01	3.34	15.71	238

Age group 51-70				
SOT	WT	α	β	count
23.51	7.85	2.49	13.41	274
21.19	6.89	3.13	13.87	327
0.82	9.71	12.68	3.36	188
2.66	12.75	12.48	4.11	51
20	5.18	3.43	14.52	220
20.98	7.85	3.01	14.31	241
21.55	8	2.77	13.5	228
22.35	10.78	3.29	13.09	104

Age group 71-80				
SOT	WT	α	β	count
21.14	7.49	2.42	11.54	89
0.84	9.98	10.44	2.7	46
20.65	8.72	2.75	11.41	105
19.89	6.79	2.59	11.59	52
23.51	7.96	1.92	11.61	93
21.91	7.81	2.67	11.18	84
22.94	9.23	2.64	10.42	90
20.16	5.25	2.43	11.27	69

We further calculate several critical properties for each cluster center. Namely:

• *Winding down period* is defined as the period of time during which $\frac{d\left[activity\left(t\right)\right]}{dt}<0$ . We calculate this quantity as $\int_{t\in \frac{d\left(activity\right)}{dt}<0}tdt$

• *Winding down activity* is defined as the overall activity level during the winding-down period. This is calculated as $\int_{t\in \frac{d\left(activity\right)}{dt}<0}activity\left(t\right)dt$

• *Overall activity* is defined as the overall activity level of the subject. This is calculated as $\int_{0}^{24}activity\left(t\right)dt$

**FIGURE 3:**
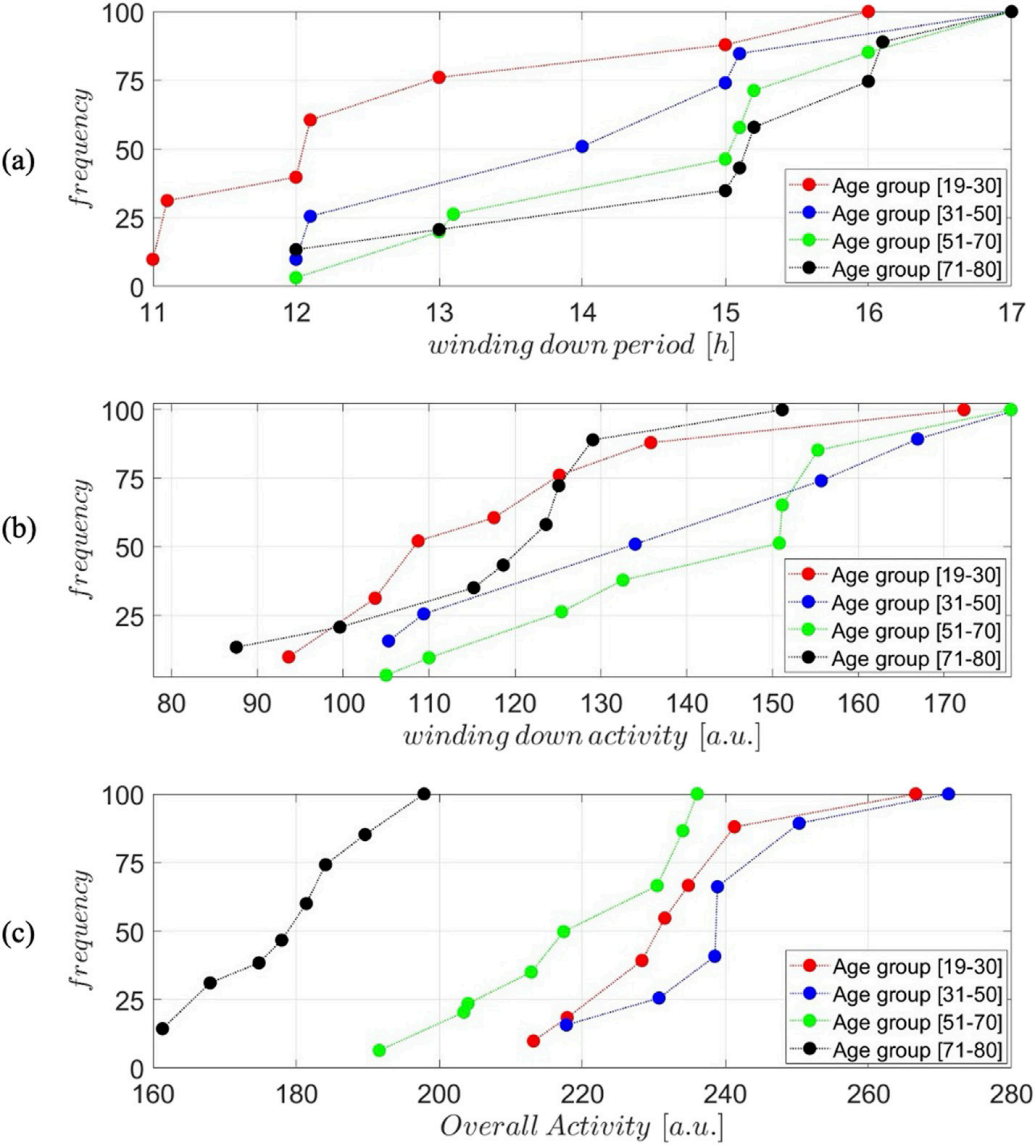
Cumulative frequency distributions of different metrics across age groups. **(A)** The distribution of the winding down period (in hours) for four age groups: 19–30, 31–50, 51–70, and 71–80. **(B)** Cumulative distribution of winding down activity levels (arbitrary units, a.u.) across the same age groups. **(C)** Cumulative distribution of overall activity levels (arbitrary units, a.u.) for the age groups. The analysis reveals distinct trends across the metrics, reflecting variations in behavior patterns between the age groups. The Wasserstein Distance test was implemented to confirm the statistical significance of the differences between the curves.

The three metrics above for all cluster centers are depicted in Figure 3 for all age groups in the form of cumulative frequency distributions. In other words, we depict the fraction of a cluster’s population and the corresponding value for each metric. The Wasserstein Distance test was implemented to confirm the statistical significance of the differences between the curves which quantifies the “cost” of transforming one distribution into another by considering the cumulative differences across the entire distribution, weighted by the distance the “mass” is shifted. It provides a more intuitive measure of overall distributional shifts and differences in shape, location, or scale.

We further examined the time it takes for the individual represented by the cluster center to reach the start of its peak performance. We define this term (Equation 2) as *time to alertness* and it is calculated as:

$$TtA={t_{\left\{\max\left(\frac{d\left(activity\right)}{dt}\right)\right\}}-t}_{\left\{\frac{d\left(activity\right)}{dt}=0\right\}}$$ (2)

**FIGURE 4:**
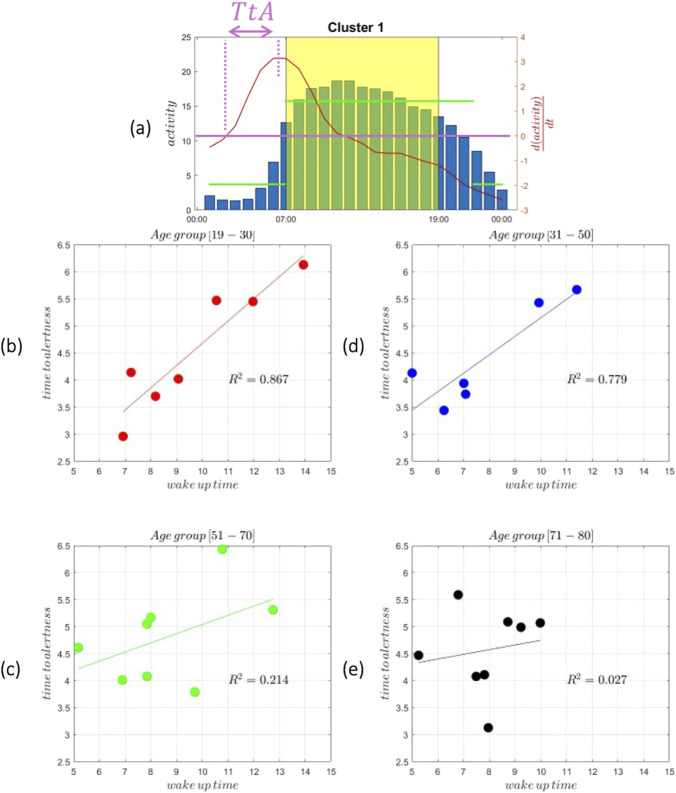
**(a)** Typical cluster center distribution of activity levels throughout the day with the shaded yellow region highlighting the waking period. TtA (time to alertness) is marked as the time interval between waking up and reaching a defined activity threshold defined as $TtA={t_{\left\{\max\left(\frac{d\left(activity\right)}{dt}\right)\right\}}-t}_{\left\{\frac{d\left(activity\right)}{dt}=0\right\}}$ . The histogram (blue bars) is overlaid with the activity curve (red line) and its derivative (Bottom) Scatter plots of wake-up time versus time to alertness ( TtA ) across four age groups: **(b)** 19–30, **(c)** 31–50, **(d)** 51–70, and **(e)** 71–80. Linear regression of wake-up time versus time-to-alertness across age groups. Significant positive associations were observed in younger adults (19–30 years, $R^{2}=0.867$ , p = 0.0023; 31–50 years, $R^{2}=0.779$ , p = 0.0199), while no significant associations were detected in older adults (51–70 years, $R^{2}=0.214$ , p = 0.248; 71–80 years, $R^{2}=0.027$ , p = 0.697).

**FIGURE 5:**
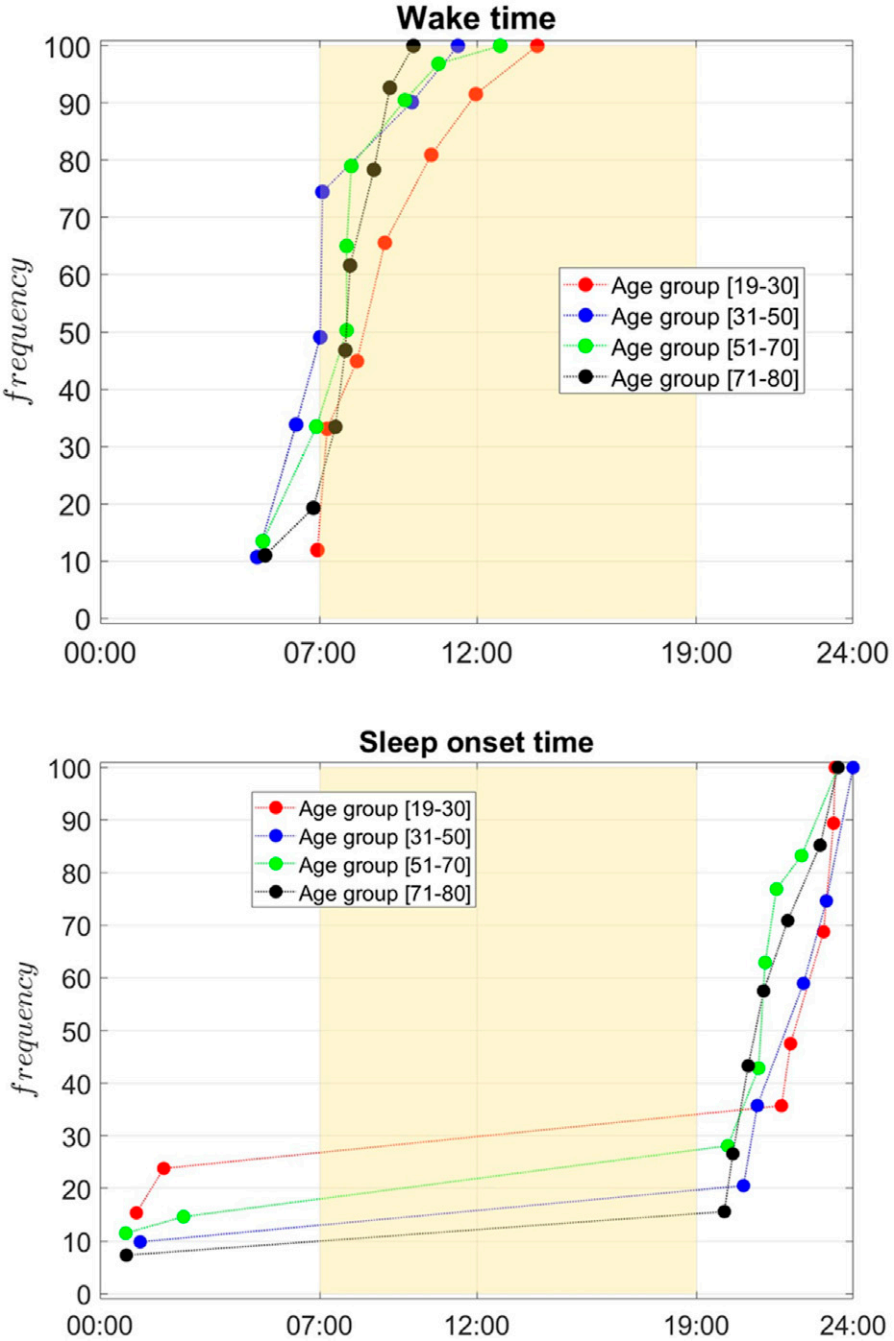
Cumulative frequency distribution of wake times (top) and sleep onset times (bottom) across age groups: 19–30 (red), 31–50 (blue), 51–70 (green), and 71–80 (black). The yellow region marks typical daytime (07:00–19:00). Younger groups show later sleep onset and wake times, with curves shifting earlier as age increases. Older groups (51–70 and 71–80) predominantly wake earlier and go to bed earlier, reflecting age-related shifts in circadian rhythms. The Wasserstein Distance test was implemented to confirm the statistical significance of the differences between the curves.

TtA is defined as the time from when the derivative of the activity is 0, which denotes a transition from inactive to active, to the time when the derivative of the activity reaches its peak; that is, the time derivative of the activity is 0. Figure 4 depicts TtA for all cluster centers and all age groups (the insert on the left depicts the definition). The weighted cumulative distribution of SOT and WT across age groups is shown in Figure 5. Finally, in the form of polar plots we depict the sleep onset (filled circles) and wake times (open circles) across four age groups, with circle sizes proportional to the frequency of each timing. These results are used to examine the progressive shift of sleep onset and wake times with increasing age, along with examining sleep duration changes across age groups.

## Discussion

We analyzed actigraphy data collected over 7 days to determine the predominant characteristics in the recorded activity. Figure 1a presents an example (NHANES SEQN 67096) of how the original minute-by-minute activity data are visualized, showing variations in activity throughout the 7-day period. This raw representation provides an overview of the subject’s activity. To identify periodicity, we compute the autocorrelation function (ACF), which measures the similarity of the activity time series at different time lags. This analysis is critical for detecting repeating patterns in the data, such as the circadian rhythm. The autocorrelation is computed for lags up to 168 h (7 days). The ACF values are smoothed to reduce noise, and peaks in the function are detected. These peaks represent time intervals at which activity patterns repeat. Figure 1d shows the resulting autocorrelation function, where clear periodic peaks occur around the 24-h mark, indicating the presence of a circadian rhythm. The time intervals between successive peaks are calculated from the detected peaks to estimate the dominant periodicity. A statistical $\chi^{2}$ test is applied to compare the observed periods against the expected 24-h rhythm. The null hypothesis assumes that the periodicity is centered around 24 h. If the $p_{val}$ exceeds a predefined threshold, the data are considered consistent with a periodic pattern. If the $p_{val}$ falls below the threshold, the periodicity is deemed inconsistent. This classification determines whether the file is accepted or rejected for further circadian analysis.

Our analysis has focused on identifying actograms reflecting a 24-h periodicity. Clearly, this is a hypothesis/limitation. However, it does not take away from the analysis. Having access to only 7 days of data are somewhat limiting in determining with confidence whether multiple patterns exist within one sequence of recordings. Therefore, we limit ourselves to actograms exhibiting robust periodicity, realizing we have omitted other patterns. A wealth of information is generated and needs to be better understood. Figure 1b is an hourly averaged representation of the activity data, simplifying daily patterns’ visualization. Peaks and troughs in this panel reflect the subject’s daily activity cycles, offering insights into the circadian structure. Figure 1c represents the average, per min, activity across all days (green line, $Y\left(t\right),t=1,\ldots ,24\times 60$ ). This represents the “average day” combining the recording from all days. This average representation is subsequently represented on an hourly basis by average consecutive 60 min elements to express an average hourly activity of each individual (red line, $y_{pa}\left(\tau \right),\tau =1,\ldots ,24$ ). Averaging activity recordings over 7 days to generate a representation of an “average day” can be justified when examining circadian behavior patterns (Shim et al., 2023). Averaging data over 7 days enhances reliability by minimizing daily fluctuations from external factors, highlighting stable trends in activity and rest. It improves consistency, reduces outliers’ impact, and facilitates clustering and comparability across individuals. This approach effectively captures habitual rhythms, offering a clearer depiction of typical behavior. However, it may obscure irregular routines, short-term fluctuations, or disruptions like insomnia, potentially masking meaningful variability. While it improves robustness and standardization, it cannot distinguish between true circadian rhythms and misalignments, limiting sensitivity to subtle changes in activity timing and intensity.

Our analysis considered each age group separately. Unlikely earlier works (Shim et al., 2023) we wanted to focus on elucidating age-dependent characteristics. Although an integrated analysis has also been performed (results not shown), the aggregation of age group obscures the analysis. As such, we considered four age groups, as explained earlier: [age group 19–30], [age group 31–50], [age group 51–70], [age group 71–80]. Each group’s actograms were clustered separately and the clusters for all groups are shown in Figure 2. The graphs depict:

a. The hourly activity levels for each cluster’s center (bars);
b. The numerical calculated derivative of the activity levels (red line); and finally,
c. the piece-wise constant approximation (green line).

The latter is used to develop a general estimate of the SOT and WT proxy. By considering the clusters, several informative observations can be made. First of all, it must be noted that cluster dynamics are relatively common across all age groups. In other words, the age-dependent differences are somewhat subtle, and it is hard to assign unique characteristics only based on age. Furthermore, by considering the activity level it is generally observed that the level of activity, looking at the maximum levels of activity, overall drops as age increases. However, it is hard to develop clearer insights when considering the form of emerging dynamics per age group. Further analysis of this data begins to unravel interesting differentiating characteristics.

Figure 3 provides a detailed comparison of actigraphy-derived sleep and activity metrics across all age groups. All distributions were determined to be statistically different from each other. The metrics are depicted in the form of CDF represented by the fraction of individuals (within a cluster) that have been assigned a specific property value. Figure 3 (top panel) explores the winding down period, defined as the duration where activity decreases (negative derivative), $\int tdt:t\in \frac{d\left(activity\right)}{dt}<0$ , and reveals a slight increase in its length with age. The winding down period aims at determining the period of time during which activity level are maintained. This is determined by evaluating the derivative of the activity: positive derivatives indicate period of increased activity, near zero derivatives indicate constancy in activity, whereas negative derivatives indicate a slowing down of activity. Younger individuals [age group 19–30] show a shorter winding down period, consistent evidence supporting an increase in sleep latency with age (Mander et al., 2017). In older groups, especially the [age group 71–80] cohort, the winding down period becomes longer, suggesting that the in older population there is a protracted period of slowing down, or in other words the length of time during which activity remains high is reduced.

Figure 3 (middle panel) quantifies winding down activity, defined as the integral of activity during periods of slow down, $\int activity\left(t\right)dt;t\in \frac{d\left(activity\right)}{dt}<0$ . Younger individuals show higher winding down activity, which could indicate more pronounced evening behaviors such as physical or social activities before sleep. As age increases, winding down activity decreases, particularly in the [age group 71–80] group, suggesting lower levels of evening activity and a more gradual, subdued decline in activity leading up to sleep. What this implies is that in younger individuals the winding down period may be short, expressed in time units, however, younger individuals remain relatively more active even during their slowing down period.

Figure 3 (bottom panel) examines overall activity during the day, showing a decrease with age. The [age group 31–50] age group exhibits the highest levels of activity, with values decreasing progressively across age groups, culminating in the lowest overall activity in the [age group 71–80] cohort, consistent with prior observations (Milanovic et al., 2013). This decline reflects the natural reduction in physical activity associated with aging, likely influenced by reduced mobility, health-related limitations, and lifestyle changes. Notably, the variability in overall activity is higher in younger groups, likely reflecting a mix of active and sedentary individuals, whereas older groups show more uniform patterns of lower activity. Together, these findings highlight the progressive changes in sleep-wake dynamics and physical activity with age: younger individuals display later wake times, shorter transitions to sleep, and higher overall activity, while older adults experience earlier wake times, extended winding down periods, and reduced physical activity, reflecting age-related biological and behavioral adaptations.

Figure 4 examines how time to alertness (TtA) varies with age, and the results are particularly interesting. As earlier noted, we define (Equation 3) the time to alertness as

$$TtA={t_{\left\{\max\left(\frac{d\left(activity\right)}{dt}\right)\right\}}-t}_{\left\{\frac{d\left(activity\right)}{dt}=0\right\}}$$ (3)

In younger adults [age group 19–30], the results demonstrate a robust positive relationship between wake-up time and time to alertness, with an $R^{2}=0.867$ . This strong correlation reflects the heightened sensitivity of this age group to circadian phase shifts. During adolescence and early adulthood, individuals typically experience a delayed circadian phase (Gradisar and Crowley, 2013), leading to a preference for later bedtimes and wake-up times, commonly referred to as an “evening chronotype.” This delay in biological rhythms peaks around the early 20 s and begins to shift earlier with age. Consequently, when young adults wake up later than usual, the misalignment of their biological clock amplifies sleep inertia, the grogginess experienced after waking, resulting in a longer time to reach full alertness. This group is also prone to accumulating sleep debt due to inconsistent schedules, social obligations, and external demands such as school and work. The variability in wake-up time can exacerbate social jetlag, a condition where the internal body clock misaligns with socially imposed schedules. These disruptions to the sleep-wake cycle impact how quickly alertness can be regained after waking. For middle-aged adults aged [age group 31–50], the relationship between wake-up time and time to alertness remains positive, though weaker, with an $R^{2}=0.779$ . As individuals move into their 30s and 40 s, the circadian rhythm becomes more stable and begins to advance slightly, favoring earlier wake-up times, a trend known as a “morning chronotype.” This stability, coupled with structured sleep patterns often imposed by work and family responsibilities, reduces the variability in sleep schedules compared to younger adults. While sleep deprivation can still impair alertness, the resilience of this age group to circadian disruptions increases. Moreover, their reduced sensitivity to phase delays means that later wake-up times have a slightly smaller impact on time to alertness than in younger individuals. However, sleep inertia persists in middle age, indicating that regular wake-up times remain important for optimizing morning alertness. In adults aged [age group 51–70], the relationship between wake-up time and time to alertness weakens considerably, with an $R^{2}=0.214$ . This reflects a shift in both biological and behavioral patterns. As people age, their circadian rhythms advance, leading to earlier bedtimes and wake-up times. This phase advance is accompanied by a reduction in the amplitude of the circadian rhythm, meaning that the signals governing the sleep-wake cycle become weaker. Additionally, sleep architecture undergoes changes with age: slow-wave sleep (deep sleep) decreases, REM sleep may shorten, and overall sleep becomes lighter and more fragmented. These changes contribute to poorer sleep efficiency, even though wake-up times tend to become more consistent due to habitual routines and fewer external demands. The diminished impact of wake-up time on time to alertness can be attributed to these more predictable schedules and the reduced ability of older adults to extend sleep duration, regardless of wake-up time. The weaker correlation suggests that factors beyond wake-up time, such as sleep quality, physical health, and medication use, may be more prominent in determining alertness in this age group. In older adults [age group 71–80], the correlation between wake-up time and time to alertness nearly disappears, with an $R^{2}=0.027$ . This finding is consistent with the literature, loss of circadian rhythmicity with age (Hood and Amir, 2017). The amplitude of circadian oscillations weakens, reducing the body’s responsiveness to changes in sleep timing or external cues such as light exposure. Elderly adults often exhibit short, fragmented sleep due to reduced homeostatic sleep drive and age-related changes in the brain’s ability to regulate sleep. Consequently, time to alertness becomes less dependent on wake-up time and may instead reflect other factors such as health conditions, medications, or daytime activity levels. Additionally, this age group often maintains consistent wake-up schedules driven by biological changes and behavioral routines, minimizing variability in time to alertness. As seen in the actigraphy results, the relationship between wake-up time and time to alertness varies across age groups. The relationship between aging and sleep inertia is very complex and hard to decipher in detail and is known to exhibit substantial variability (Ruby et al., 2024; Tonet et al., 2022). The results highlight a strong correlation between wake-up time and time to alertness in younger age groups, a gradually diminishing trend in middle-aged adults, and a near absence of any relationship in older adults. These patterns are likely deeply rooted in both biological and behavioral changes across the human lifespan.

Figure 5 illustrates the cumulative frequency distribution of wake times (top panel) and sleep onset times (bottom panel) (determined based on the piece-wise constant approximation) across four age groups: [19–30] (red), [31–50] (blue), [51–70] (green), and [71–80] (black). Overall sleep onset and offset are generally calculated based on scoring rules that use a predefined number of consecutive minutes over which the individual is at rest or exhibits movement following rest (Fekedulegn et al., 2020). In our case, these were determined based on the fitted parameters of each cluster’s center. The yellow-shaded region represents the typical daytime period from 07:00 to 19:00, providing a visual reference for the alignment of sleep-wake patterns with the conventional day-night cycle. Younger age groups, particularly those aged [19–30], exhibit a wake time distribution that peaks substantially later in the day, with the curve rising steeply in the late morning. The [31–50] age group shows a shift toward earlier waking, the [51–70] and [71–80] display similar wake times, flanked between the two other groups. This progression suggests a clear trend toward earlier wake times with increasing age. A similar pattern is observed for sleep onset times. The youngest group [19–30] demonstrates a later sleep onset, with a higher frequency of individuals going to bed closer to midnight. The 31–50 group follows a similar trend but with slightly earlier sleep onset times compared to the youngest cohort. Among individuals aged [51–70], sleep onset times are predominantly earlier in the evening, peaking between 9:00 p.m. and 10:00 p.m. For the oldest group (71–80), sleep onset times shift earlier, with most individuals going to bed well before midnight. These observations collectively highlight the impact of age on sleep-wake patterns, with older individuals tending to wake up and go to bed earlier, reflecting age-related changes in circadian rhythms. Conversely, younger adults exhibit a preference for later wake times and sleep onset, consistent with delayed sleep-wake preferences typically associated with younger age groups. The results reveal interesting characteristics. WT and SOT vary widely within age groups, but also there is a general trend for moving toward an earlier chronotype, based on of WT , with aging. Wake-up time shifts earlier with increasing age, a trend that aligns with the well-documented age-related phase advance in circadian rhythms, where older adults tend to shift toward morningness (Duffy et al., 2015). The variability in wake-up time is greater in younger age groups, likely due to irregular sleep schedules influenced by lifestyle factors such as work, study, and social activities. In contrast, wake-up times in older adults are more consistent, reflecting a greater regularity in daily routines and biological changes that reduce sleep duration.

**FIGURE 6:**
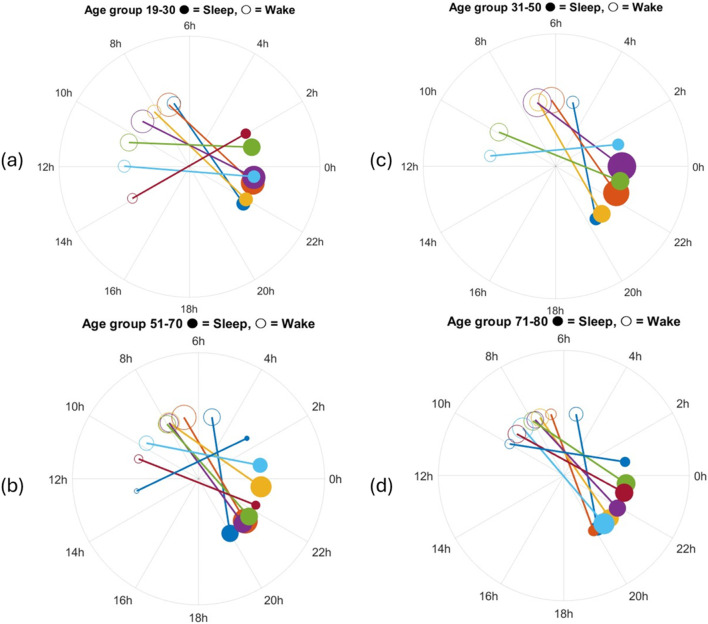
Polar plots showing sleep onset (filled circles) and wake times (open circles) for individuals across four age groups: **(a)** 19–30, **(b)** 51–70, **(c)** 31–50, **(d)** 71–80. The connecting lines represent the duration of individual sleep periods. Circle sizes are proportional to the frequency of occurrence for each sleep-wake time. Younger age groups (19–30, 31–50) exhibit later sleep onset and wake times, with longer sleep durations, while older age groups (51–70, 71–80) display earlier sleep onset and wake times, reflecting age-related shifts in sleep timing and duration. The circle sizes are proportional to the size of the corresponding cluster.

Figure 6 illustrates, in the form of polar plots, the distribution of sleep onset times (filled circles) and wake times (open circles) for the four age groups with lines connecting the two times for each cluster. The size of each circle represents the frequency of a particular sleep-wake combination within an age group based on the number of subjects assigned to that cluster. The [19–30] age group demonstrates a clear preference for later sleep onset and wake times, with most individuals going to bed close to midnight and waking up around 7–9 a.m. This group shows longer sleep durations on average. The [31–50] group follows a similar pattern, with slightly earlier sleep onset and wake times compared to the [19–30] group, but still within a later timing preference compared to older groups. In contrast, the [51–70] and [71–80] groups exhibit distinctly earlier sleep-wake patterns. The [51–70] group shows a clustering of sleep onset around 10 p.m. and wake times close to 5–7 a.m., suggesting a shortened sleep duration compared to younger groups. The [71–80] group displays an even more pronounced shift toward early sleep onset (around 9–10 p.m.) and early wake times (4–6 a.m.), with many individuals showing compressed sleep periods as reported in the literature (Li et al., 2018). These plots highlight the progressive shift in sleep timing and duration with increasing age, reflecting well-documented age-related changes in circadian rhythms. Younger groups tend to exhibit later sleep-wake preferences and longer sleep durations, while older groups display a trend toward earlier sleep-wake cycles and shorter durations. The variation in circle sizes further emphasizes the higher frequency of early sleep onset and wake times in older groups, underscoring a consistent pattern of advancing sleep phase with age.

This study underscores actigraphy’s value in large-scale research, providing real-world insights into the interaction between behavior, environment, and biological rhythms. The findings highlight wearable technology’s potential to advance personalized healthcare and public health strategies. Addressing knowledge gaps on irregular routines and circadian variations can inform policies that support healthy aging. Ultimately, leveraging activity patterns and circadian rhythms can improve health outcomes, reduce lifestyle-related disease burdens, and promote long-term wellbeing across populations.

Our findings suggest that age-related shifts toward earlier phase, reduced amplitude, and prolonged transition kinetics can be interpreted as remodeling of the multi-loop sleep–wake control system. Because the markers are transparent and device-independent, they can serve as observables for calibrating multiscale models (e.g., circadian–homeostatic ODEs coupled to endocrine/inflammatory axes) and for testing how light schedules, activity prescriptions, or pharmacologic interventions reshape network parameters *in silico* before clinical deployment.

In summary, a simple, transparent unsupervised approach applied to public, device-independent NHANES MIMS data recovers known age-related phase advance and declining activity and introduces actionable markers—winding-down time/activity and time-to-alertness—with age-specific normative distributions. These interpretable phenotypes complement traditional indices and can support clinical counseling, population surveillance, and evaluation of interventions targeting sleep–wake alignment across the adult lifespan.

## Data Availability

Publicly available datasets were analyzed in this study. This data can be found here: NHANES Database https://www.cdc.gov/nchs/nhanes/index.html.

## Footnotes

1
https://wwwn.cdc.gov/nchs/nhanes/search/datapage.aspx?Component=Examination&CycleBeginYear=2011

2
https://wwwn.cdc.gov/nchs/nhanes/search/datapage.aspx?Component=Examination&CycleBeginYear=2013

## Abbreviations

ACF, Autocorrelation function; PCA, Piece-wise constant Approximation; TtA, Time to alertness; SOT, Sleep onset time; WT, Wake time.
